# A large-scale systematic survey reveals recurring molecular features of public antibody responses to SARS-CoV-2

**DOI:** 10.1016/j.immuni.2022.03.019

**Published:** 2022-06-14

**Authors:** Yiquan Wang, Meng Yuan, Huibin Lv, Jian Peng, Ian A. Wilson, Nicholas C. Wu

**Affiliations:** 1 Department of Biochemistry, University of Illinois at Urbana-Champaign, Urbana, IL 61801, USA; 2 Department of Integrative Structural and Computational Biology, The Scripps Research Institute, La Jolla, CA 92037, USA; 3 HKU-Pasteur Research Pole, School of Public Health, Li Ka Shing Faculty of Medicine, The University of Hong Kong, Hong Kong SAR, China; 4 Department of Computer Science, University of Illinois at Urbana-Champaign, Urbana, IL 61801, USA; 5 The Skaggs Institute for Chemical Biology, The Scripps Research Institute, La Jolla, CA 92037, USA; 6 Center for Biophysics and Quantitative Biology, University of Illinois at Urbana-Champaign, Urbana, IL 61801, USA; 7 Carl R. Woese Institute for Genomic Biology, University of Illinois at Urbana-Champaign, Urbana, IL 61801, USA; 8 Carle Illinois College of Medicine, University of Illinois at Urbana-Champaign, Urbana, IL 61801, USA

**Keywords:** COVID-19, SARS-CoV-2, antibody, public antibody response, data mining, sequence analysis, deep learning, structural analysis, affinity maturation, somatic hypermutation

## Abstract

Global research to combat the COVID-19 pandemic has led to the isolation and characterization of thousands of human antibodies to the SARS-CoV-2 spike protein, providing an unprecedented opportunity to study the antibody response to a single antigen. Using the information derived from 88 research publications and 13 patents, we assembled a dataset of ∼8,000 human antibodies to the SARS-CoV-2 spike protein from >200 donors. By analyzing immunoglobulin V and D gene usages, complementarity-determining region H3 sequences, and somatic hypermutations, we demonstrated that the common (public) responses to different domains of the spike protein were quite different. We further used these sequences to train a deep-learning model to accurately distinguish between the human antibodies to SARS-CoV-2 spike protein and those to influenza hemagglutinin protein. Overall, this study provides an informative resource for antibody research and enhances our molecular understanding of public antibody responses.

## Highlights

• Assembled a dataset of ∼8,000 published antibodies to SARS-CoV-2 S from >200 donors
• Antibodies to RBD, NTD, and S2 have distinct convergent sequence and molecular features
• Public antibody clonotypes show recurring affinity maturation pathway
• Provided a proof of concept for antibody specificity prediction using deep learning

## Graphical abstract

**Figure:**
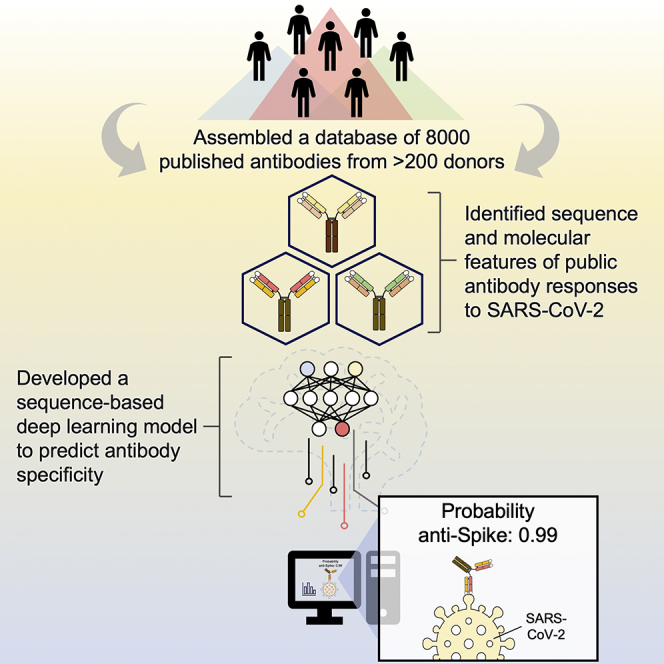

## Teaser

Since the start of the COVID-19 pandemic, the isolation of antibodies to SARS-CoV-2 spike protein has been a major research focus. Wang et al. analyzed ∼8,000 published human monoclonal antibodies to the spike protein and identified sequence and molecular features of the public antibody responses to SARS-CoV-2. The results enable the construction of a sequence-based, deep-learning model to predict antibody specificity.

## Introduction

From the beginning of the COVID-19 pandemic, many research groups worldwide turned their attention to SARS-CoV-2 and, in particular, to the immune response to infection and vaccination. Since 2020, thousands of human monoclonal antibodies to SARS-CoV-2 have been isolated and characterized (Li et al., 2022a; Raybould et al., 2021). The major surface antigen to which antibodies are elicited is the SARS-CoV-2 spike (S) protein, which is a homotrimeric glycoprotein that facilitates virus entry by first engaging the host receptor angiotensin-converting enzyme 2 (ACE2) and then mediating membrane fusion (Shang et al., 2020; Zhou et al., 2020). The S protein has three major domains, namely the N-terminal domain (NTD), receptor-binding domain (RBD), and S2 domain (Walls et al., 2020; Wrapp et al., 2020). Most studies on SARS-CoV-2 antibodies have focused on the immunodominant RBD (Yuan et al., 2021) because neutralizing antibodies can be elicited to it with very high potency (Tortorici et al., 2020; Wang et al., 2021). Antibodies to the NTD and the highly conserved S2 domain have also been discovered (Cerutti et al., 2021; Chi et al., 2020; Li et al., 2021a; 2022b; Pinto et al., 2021; Voss et al., 2021; Zhou et al., 2022b).

A common or public antibody response describes antibodies to the same antigen in different donors that share genetic elements that usually result in similar modes of antigen recognition. Deciphering public responses to particular antigens is not only critical for uncovering the molecular features of recurring antibodies within the diverse antibody repertoire at the population level, but also important for development of effective vaccines (Andrews and McDermott, 2018; Lanzavecchia et al., 2016). A conventional approach to study public antibody responses is to identify public clonotypes, which are antibodies from different donors that share the same immunoglobulin-heavy variable (IGHV) gene and with similar complementarity-determining region (CDR) H3 sequences (Henry Dunand and Wilson, 2015; Jackson et al., 2014; Pieper et al., 2017; Setliff et al., 2018; Trück et al., 2015). While this definition of public clonotypes has improved our understanding of public antibody response, it generally ignores the contribution of the light chain. Moreover, our recent study has shown that a public antibody response to influenza hemagglutinin (HA) is driven by an IGHD gene with minimal dependence on the IGHV gene (Wu et al., 2018). Therefore, the true extent and molecular characterization of public antibody responses remain to be explored.

Although information of many human monoclonal antibodies to SARS-CoV-2 is now publicly available, it has been difficult to leverage all available information to investigate public antibody responses to SARS-CoV-2. One major challenge is that the data from different studies are rarely in the same format. This inconsistency imposes a huge barrier to data mining. The establishment of the coronavirus antibody database (CoV-AbDab) has enabled researchers to deposit their antibody data in a standardized format and has partially resolved the data formatting issue (Raybould et al., 2021). However, not every SARS-CoV-2 antibody study has deposited their data to CoV-AbDab. Furthermore, IGHD gene identities, nucleotide sequences, and donor IDs are not available in CoV-AbDab, which makes it challenging to study public antibody responses using CoV-AbDab. Thus, additional efforts must be made to fully synergize the information across many different SARS-CoV-2 antibody studies to investigate and decipher public antibody responses.

In this study, we performed a systematic literature survey and assembled a large dataset of human SARS-CoV-2 monoclonal antibodies with donor information. We then analyzed this dataset and uncovered many antibody sequence features that contribute to the public antibody responses to SARS-CoV-2 S. For example, we identified a public antibody response to RBD that is largely independent of the IGHV gene, as well as involvement of a particular IGHD gene in a public antibody response to S2. Our analysis also revealed a number of recurring somatic hypermutations (SHMs) in different public clonotypes. All of these sequence features provide a foundation for using deep learning to identify SARS-CoV-2 S antibodies.

## Results

### A large-scale collection of SARS-CoV-2 antibody information

Information for 8,048 human antibodies was collected from 88 research publications and 13 patents that described the discovery and characterization of antibodies to SARS-CoV-2 (Figure S1; Table S1). Among these antibodies, which were isolated from 215 different donors, 7,997 (99.4%) react with SARS-CoV-2, and the remaining 51 react with SARS-CoV or seasonal coronaviruses. While 99.1% (7,923/7,997) SARS-CoV-2 antibodies in our dataset bind to S protein, 49 bind to N and 25 to ORF8. Epitope information was available for most SARS-CoV-2 S antibodies, with 5,002 to RBD, 513 to NTD, and 890 to S2. In addition, information on neutralization activity, germline gene usage, sequence, structure, bait for isolation (e.g., RBD and S), and donor status (e.g., infected patient, vaccinee, etc.), if available, was collected for individual antibodies. Using this large dataset, we aimed to analyze the sequence features of public antibody responses to SARS-CoV-2 S.

### Antibodies to RBD, NTD, and S2 have distinct V gene usage bias

**Figure 1:**
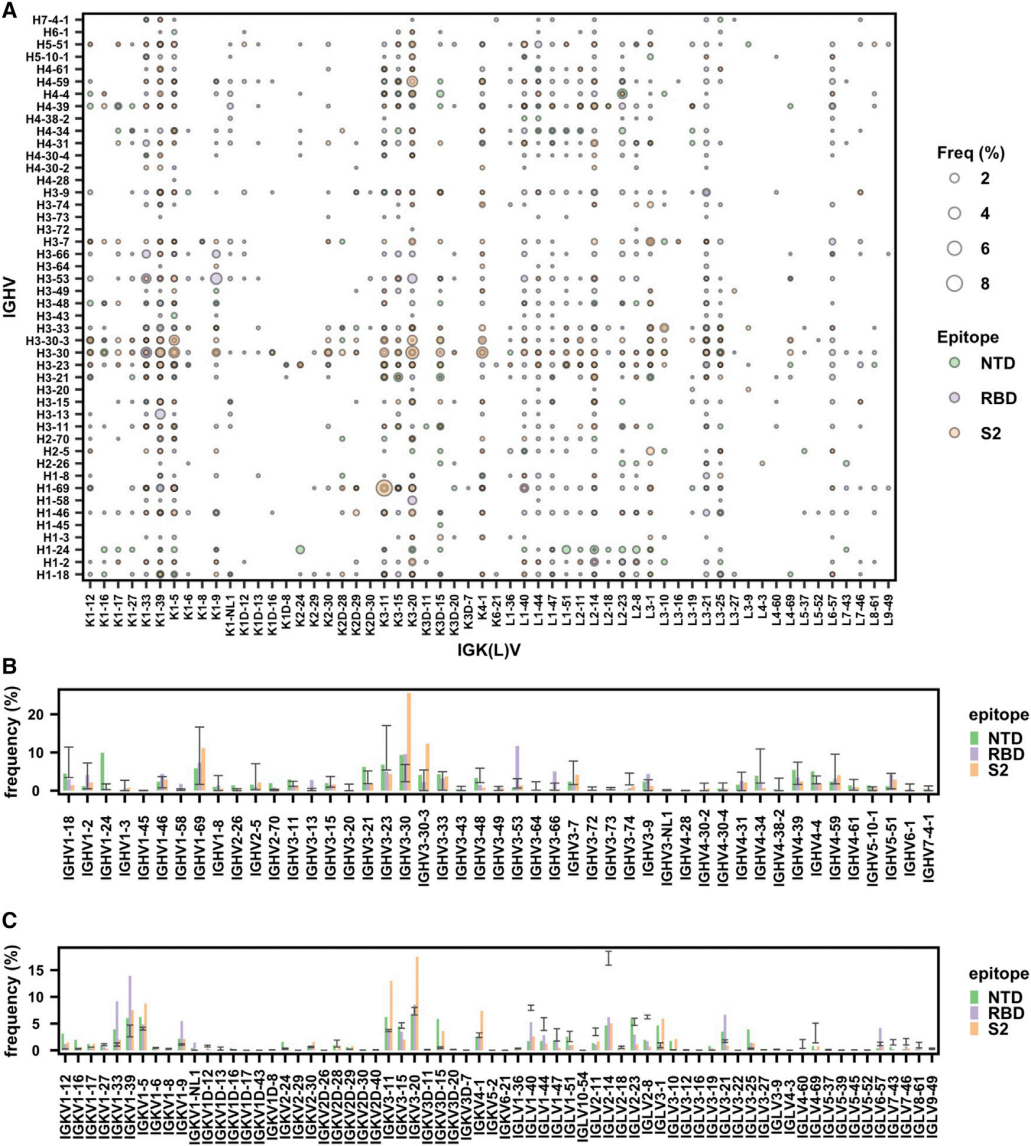
Antibodies to different domains of SARS-CoV-2 S have distinct patterns of V gene usage (A) The frequency of different V gene pairings between heavy and light chains are shown for SARS-CoV-2 S antibodies to RBD, NTD, and S2. The size of each data point represents the frequency of the corresponding IGHV/IGK(L)V pair within its epitope category. Only those antibodies where both IGHV and IGK(L)V information is available for both heavy and light chains were included in this analysis. (B) The IGHV gene usage in antibodies to NTD, RBD, and S2 is shown. Only those antibodies with IGHV information available were included in this analysis. (C) The IGK(L)V gene usage in antibodies to NTD, RBD, and S2 is shown. Only those antibodies with IGK(L)V information available were included in this analysis. (B and C) Error bars represent the frequency range among 26 healthy donors (Briney et al., 2019; Guo et al., 2019; Soto et al., 2019). See also Figure S1 and Tables S1 and S2.

We first performed an analysis on the V gene usage of SARS-CoV-2 S antibodies. Our analysis captured previously known V gene usage patterns, including the prevalence of IGHV3-53/IGKV1-9 and IGHV3-53/IGKV3-20 among RBD antibodies (Cao et al., 2020; Clark et al., 2021; Kim et al., 2021; Tan et al., 2021; Yuan et al., 2020; Zhang et al., 2021; Figure 1A), as well as substantial enrichment of IGHV1-24 among NTD antibodies (Cerutti et al., 2021; Chi et al., 2020; Li et al., 2021a; Voss et al., 2021; Figure 1B). Importantly, our dataset also enabled us to discover previously unknown patterns in gene usage. For example, IGHV3-30 and IGHV3-30-3 were highly enriched among S2 antibodies (Figure 1B). V gene usage bias was also observed in the light chain. For example, IGKV3-20 and IGKV3-11 were most used among S2 antibodies, whereas IGKV1-33 and IGKV1-39 were most used among RBD antibodies (Figure 1C). Overall, these results demonstrated that RBD, NTD, and S2 antibodies have distinct patterns of V gene usage and that both heavy and light-chain V genes contribute to the public antibody response to SARS-CoV-2 S.

### CDR H3 analysis reveals domain-specific public antibody response

**Figure 2:**
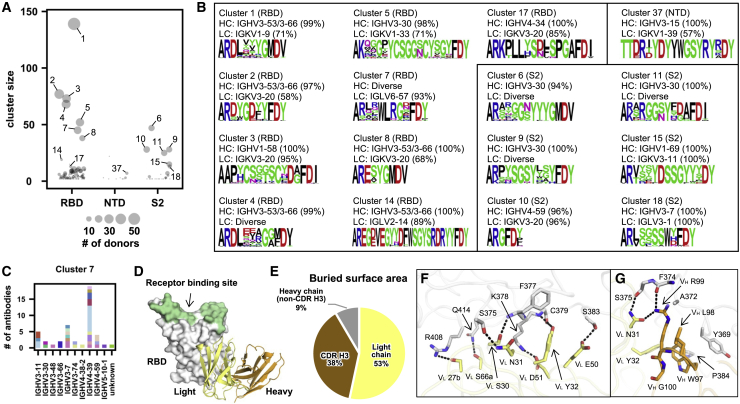
SARS-CoV-2 S antibodies exhibit convergent CDR H3 sequences (A) CDR H3 sequences from individual antibodies were clustered using a 80% sequence identity cutoff (see STAR Methods). The epitope of each CDR H3 cluster is classified based on that of its antibody members. Cluster size represents the number of antibodies within the cluster. (B) The V gene usage and CDR H3 sequence are shown for each of the 16 CDR H3 clusters of interest. For each of the CDR H3 cluster of interest, the CDR H3 sequences are shown as a sequence logo, where the height of each letter represents the frequency of the corresponding amino-acid variant (single-letter amino-acid code) at the indicated position. The dominant germline V genes (>50% usage among all antibodies within a given CDR H3 cluster) are listed. Diverse: no germline V genes had >50% frequency among all antibodies within a given CDR H3 cluster. HC, heavy chain; LC, light chain. Clusters with the same domain specificity are grouped in the same box. (C) IGHV usage in cluster 7 is shown. Different colors represent different donors. Unknown: IGHV information is not available. (D) An overall view of SARS-CoV-2 RBD in complex with IGLV6-57 antibody S2A4 (PDB 7JVA) (Piccoli et al., 2020), which belongs to cluster 7, is shown. The RBD is in white with the receptor-binding site highlighted in green. The heavy and light chains of S2A4 are in orange and yellow, respectively. (E) Percentages of the S2A4 epitope that are buried by the light chain, heavy chain (without CDR H3), and CDR H3 are shown as a pie chart. Buried surface area (BSA) was calculated by proteins, interfaces, structures, and assemblies (PISA) at the European Bioinformatics Institute (https://www.ebi.ac.uk/pdbe/prot_int/pistart.html) (Krissinel and Henrick, 2007). (F and G) Detailed interactions between the (F) light and (G) heavy chains of S2A4 and SARS-CoV-2 RBD. Hydrogen bonds and salt bridges are represented by black dashed lines. The color coding is the same as (D). See also Figures S2–S4 and Tables S1 and S2.

Most of the antibody sequence diversity comes from the CDR H3 region due to V(D)J recombination (Elhanati et al., 2015; Jung and Alt, 2004; Schatz and Swanson, 2011). To identify the sequence features of CDR H3 in public antibody response to SARS-CoV-2 S, CDR H3 sequences with the same length were clustered by an 80% sequence identity cutoff. A total of 170 clusters that contained antibodies from at least two different donors were identified (Figure 2A; Table S1).

Most of the antibodies in the largest cluster (cluster 1, Figure 2B) belonged to a well-characterized public clonotype to RBD that is encoded by IGHV3-53/3-66 and IGKV1-9 (Cao et al., 2020; Clark et al., 2021; Kim et al., 2021; Tan et al., 2021; Zhang et al., 2021). IGHV3-53/3-66, which is frequently used in RBD antibodies (Yuan et al., 2020), was also enriched among antibodies in several other major CDR H3 clusters (e.g., clusters 2, 4, 8, and 14). Antibodies that bind to quaternary epitopes by bridging two RBDs on the same spike were found in clusters 14 and 17 (Barnes et al., 2020; Figure S2). Notably, both clusters 3 and 5, which targeted the RBD, contained a pair of highly conserved cysteines, suggesting the presence of a disulfide bond within the CDR H3 (Figure 2B). Cluster 3 represented another well-characterized public clonotype that is encoded by IGHV1-58/IGKV3-20 and indeed contains an intra-CDR H3 disulfide bond (Dejnirattisai et al., 2021; Robbiani et al., 2020; Tortorici et al., 2020; Wang et al., 2021; Reincke et al., 2022). On the other hand, antibodies in cluster 5, which were largely encoded by IGHV3-30/IGKV1-33, have not been extensively studied. Most antibodies within cluster 5 had relatively weak neutralizing activity, if any, despite having reasonable binding affinity (Table S2). This result suggests the existence of an RBD-targeting public clonotype that had minimal neutralizing activity. Similar observation was made with RBD antibodies encoded by IGHV3-13/IGKV1-39, although most of these antibodies did not share a similar CDR H3 (Figure S3; Table S2). The weak neutralizing activity of these antibodies may at least be partly attributed to their inability to compete with ACE2, as demonstrated by the structural studies of the IGHV3-30/IGKV1-33-encoded RBD antibody COVOX-45 (Dejnirattisai et al., 2021), and the IGHV3-13/IGKV1-39-encoded RBD antibody S304 (Piccoli et al., 2020; Starr et al., 2021; Thomson et al., 2021).

Furthermore, we also discovered several S2-specific CDR H3 clusters (clusters 6, 9, and 11) that were predominantly encoded by IGHV3-30 with diverse IGK(L)V genes, suggesting a public heavy-chain response to S2 (Figure 2B). Clusters 10 and 15 were also of particular interest. Cluster 10 featured a very short CDR H3 (6 amino acids, IMGT numbering) and was encoded by IGHV4-59/IGKV3-20, which was a frequent V gene pair among the S2 antibodies (Figure 1A). Cluster 15 was encoded by IGHV1-69/IGKV3-11, which was the most used V gene pair among the S2 antibodies (Figure 1A). Therefore, clusters 10 and 15 represented two major S2 public clonotypes, despite their minimal neutralizing activity (Table S2). In contrast to RBD- and S2-specific clusters, all NTD-specific CDR H3 clusters had a relatively small size (Figure 2A), suggesting that the paratopes for most NTD antibodies are not dominated by CDR H3. Nevertheless, the small number of H3 clusters among NTD antibodies may also be due to fewer antibodies to NTD than to RBD or S2 in our dataset.

### IGLV6-57 contributes to RBD-specific public antibody response

While most clusters had a dominant IGHV gene, diverse IGHV genes were observed in cluster 7 (Figures 2B and 2C). Most antibodies (42 out of 45) in cluster 7 used IGLV6-57, suggesting their paratopes are mainly composed of CDR H3 and light chain. S2A4, which is encoded by IGHV3-7/IGLV6-57 (Piccoli et al., 2020), is an antibody in cluster 7. A previously determined structure of S2A4 in complex with RBD indeed demonstrates that its CDR H3 contributes 38% of the buried surface area (BSA) of the epitope, whereas the light chain contributes 53% (Figures 2D and 2E). Specifically, IGLV6-57 forms an extensive H-bond network with the RBD (Figure 2F), whereas a ^97^WLRG^100^ motif at the tip of CDR H3 interacts with the RBD through H-bonds, π-π stacking, and hydrophobic interactions (Figure 2G). Although G100 does not participate in binding, it exhibits backbone torsion angles (Φ = −94°, Ψ = −160°) that are in the preferred region of Ramachandran plot for glycine, but in the allowed region for non-glycine (Figure S4). Consistently, this ^97^WLRG^100^ motif is highly conserved in cluster 7 (Figure 2B). This analysis substantiates the importance of the light chain in the public antibody response to SARS-CoV-2 S.

### IGHD1-26 contributes to S2-specific public antibody response

**Figure 3:**
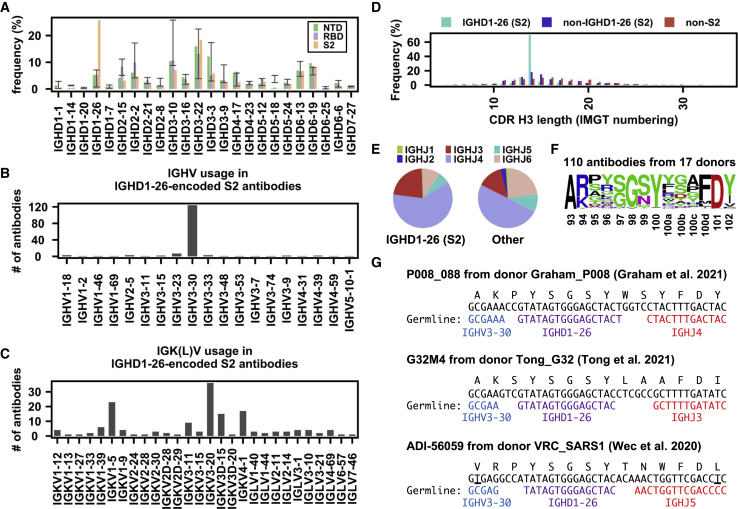
IGHD1-26 is enriched among SARS-CoV-2 S2 antibodies (A) The IGHD gene usage in NTD, RBD, S2 antibodies is shown. Error bars represent the frequency range among 26 healthy donors. (B and C) (B) IGHV gene usage and (C) IGK(L)V gene usage among IGHD1-26 S2 antibodies is shown (n = 157). (D) The distribution of CDR H3 length (IMGT numbering) in IGHD1-26 S2 antibodies (n = 157), non-IGHD1-26 S2 antibodies (n = 533), and non-S2 S antibodies (n = 5,090) are shown. (E) The IGHJ gene usage among IGHD1-26 S2 antibodies (n = 157) and other S antibodies with well-defined epitopes (n = 5,623) is shown. (F) The CDR H3 sequences for IGHD1-26 S2 antibodies (n = 110) are shown as a sequence logo. (G) Amino acid and nucleotide sequences of the V-D-J junction are shown for three IGHD1-26 S2 antibodies (Graham et al., 2021; Tong et al., 2021; Wec et al., 2020). While P008_088 and G32M4 were from SARS-CoV-2-infected individuals, ADI-56059 was from a SARS-CoV survivor. Putative germline sequences and segments were identified by IgBlast (Ye et al., 2013) and are indicated. Somatically mutated nucleotides are underlined. Intervening spaces at the V-D and D-J junctions are N-nucleotide additions. See also Tables S1 and S2.

As shown in our previous study, the IGHD gene can drive a public antibody response (Wu et al., 2018). Here, we found that IGHD1-26 was highly enriched among S2 antibodies (Figure 3A). These IGHD1-26 S2 antibodies were predominantly encoded by IGHV3-30 (Figure 3B), which was one of the most used IGHV genes among S2 antibodies (Figure 1B). In contrast, the IGK(L)V gene usage was more diverse among these IGHD1-26 S2 antibodies, although several were more frequently used than others (Figure 3C), implying that this public antibody response to S2 was mainly driven by the heavy chain. 70% of these IGHD1-26 S2 antibodies had a CDR H3 of 14 amino acids, whereas only <20% of other S antibodies had a CDR H3 of 14 amino acids (Figure 3D). In fact, most members of clusters 6, 9, and 11 in our CDR H3 analysis above (Figure 2B) represented this public antibody response to S2. While CDR H3 is also encoded by the IGHJ gene, the distribution of IGHJ gene usage in these IGHD1-26 S2 antibodies did not show a strong deviation from that of other S antibodies in our dataset (Figure 3E).

In our dataset, there were 110 IGHD1-26 S2 antibodies from 17 donors with a CDR H3 length of 14 amino acids. Most of these 110 IGHD1-26 S2 antibodies could cross-react with SARS-CoV, but with minimal neutralization activity (Table S2). Sequence logo analysis of these 110 antibodies revealed a conserved ^97^[S/G]G[S/N]Y^100^ motif in the middle of their CDR H3 sequences (Figure 3F). In-depth analysis of the CDR H3 sequences from three representative IGHD1-26 S2 antibodies from three different donors (Graham et al., 2021; Tong et al., 2021; Wec et al., 2020) further indicated that the conserved ^97^[S/G]G[S/N]Y^100^ motif was within the IGHD1-26-encoded region (Figure 3G). Together, these results show that the public antibody response to SARS-CoV-2 S also involves the IGHD gene.

### SHM analysis reveals a recurring affinity maturation pathway

**Figure 4:**
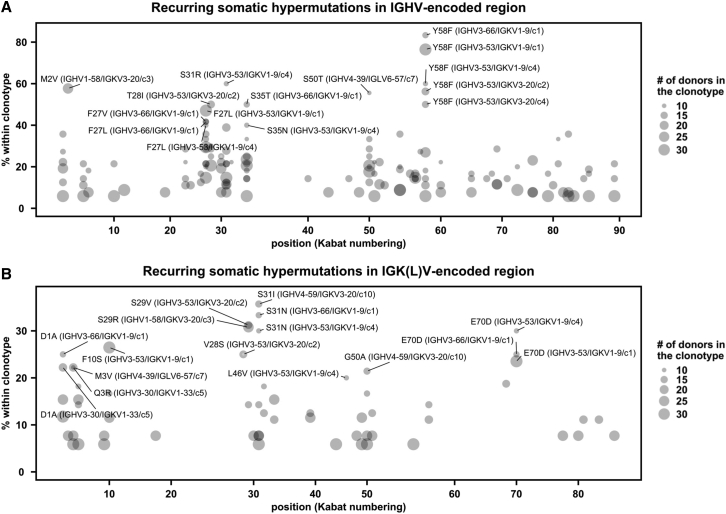
SARS-CoV-2 S antibodies contain recurring somatic hypermutations (SHMs) (A and B) For each public clonotype, if the exact same SHM emerged in at least two donors, such SHM is classified as a recurring SHM. Only those public clonotypes that can be observed in at least nine donors are shown. (A) Recurring SHMs in heavy-chain V genes. (B) Recurring SHMs in light-chain V genes. x axis represents the position on the V gene (Kabat numbering). y axis represents the percentage of donors who carry a given recurring SHM among those who carry the public clonotype of interest. For example, V_L_ S29R emerged in 8 donors out of 26 donors that carry a public clonotype that is encoded by IGHV1-58/IGKV3-20. As a result, V_L_ S29R (IGHV1-58/IGKV3-20) is 31% (8/26) within the corresponding clonotype. Of note, since each public clonotype is also defined by the similarity of CDR H3 (see STAR Methods), there could be multiple clonotypes with the same heavy- and light-chain V genes (e.g., IGHV3-53/IGKV1-9). The CDR H3 cluster ID for each clonotype is indicated with a prefix “c,” following the information of the V genes. For heavy chain, SHMs that emerged in at least 40% of the donors of the corresponding clonotype are labeled. For light chain, SHMs that emerged in at least 20% of the donors of the corresponding clonotype are labeled. See also Figure S5 and Table S1.

Our recent study has shown that V_H_ Y58F is a recurring SHM among IGHV3-53 antibodies to SARS-CoV-2 RBD (Tan et al., 2021), indicating that SHM is involved in the public antibody response to SARS-CoV-2. To identify additional recurring SHMs in SARS-CoV-2 S antibodies, antibodies from at least two donors that had the same IGHV/IGK(L)V genes and CDR H3s from the same CDR H3 cluster were classified as a public clonotype (Figure 4A). SHM that occurred in at least two donors within a public clonotypes was defined as a recurring SHM. This analysis led to the identification of several recurring SHMs in IGHV3-53/3-66-encoded public clonotypes that were previously characterized, including V_H_ F27V, T28I, and Y58F (Hurlburt et al., 2020; Scheid et al., 2021; Tan et al., 2021; Figure S5). Many of the recurring SHMs were not hotspots for activation-induced deaminase (AID) (Álvarez-Prado et al., 2018; Di Noia and Neuberger, 2007; Yeap et al., 2015). For example, among the seven recurring V_H_ SHMs that had high occurrence frequency in IGHV3-53/3-66-encoded public clonotypes (F27V, F27L, T28I, S31R, S35T, S35N, and Y58F), only V_H_ T28I and S35N involved deamination, and only V_H_ S35N was at the hotspot (nucleotide motif RGYW) for AID (Álvarez-Prado et al., 2018).

**Figure 5:**
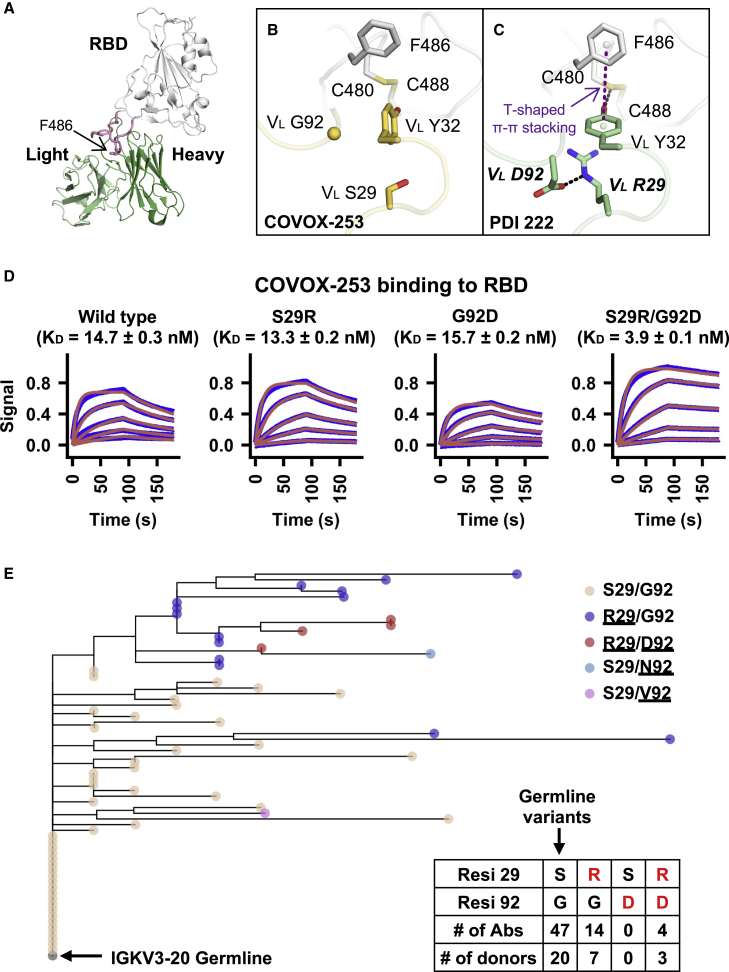
Two recurring SHMs synergistically drive the affinity maturation of a IGHV1-58/IGKV3-20 public clonotype (A) An overall view of SARS-CoV-2 RBD in complex with the IGHV1-58/IGKV3-20 antibody PDI 222 (PDB 7RR0) (Wheatley et al., 2021). The RBD is shown in white, while the heavy and light chains of the antibody are in dark and light green, respectively. The ridge region (residues 471–491) is shown in pink. (B and C) Structural comparison between two IGHV1-58/IGKV3-20 antibodies that either (B) carry germline residues V_L_ S29/G92 (COVOX-253, PDB 7BEN) (Dejnirattisai et al., 2021) and (C) somatically hypermutated residues V_L_ R29/D92 (PDI 222, PDB 7RR0) (Wheatley et al., 2021). SARS-CoV-2 RBD is in white, while antibodies are in yellow (COVOX-253) and green (PDI 222). Somatically mutated residues are labeled with bold and italic letters. The T-shaped π-π stacking between RBD-F486 and V_L_ Y32 is indicated by a purple dashed line. Hydrogen bond and salt bridge are represented by black dashed lines. (D) Binding kinetics between COVOX-253 Fabs (wild type or mutants) and SARS-CoV-2 RBD were measured by biolayer interferometry (BLI). y axis represents the response. Blue lines represent the response curves and red lines represent the 1:1 binding model. Binding kinetics were measured for five concentrations of the RBDs at 3-fold dilution ranging from 300 to 3.7 nM. The dissociation constant (K_D_) values ± standard deviations are indicated. (E) A phylogenetic tree was constructed for the light-chain sequences of 67 antibodies in the IGHV1-58/IGKV3-20 public clonotype. The phylogenetic tree was rooted using the germline sequence of IGKV3-20. Each tip represents one antibody and is colored according to the corresponding amino acid variants at V_L_ residues 29 and 92. Amino acid variants that represent SHM are underlined. Numbers of antibodies in the IGHV1-58/IGKV3-20 public clonotype carrying the germline-encoded variant at V_L_ residues 29 and 92 (S29, G92), as well as V_L_ SHM S29R and G92D (red) are listed in the inset table. Of note, one antibody in this IGHV1-58/IGKV3-20 public clonotype carries S29/N92 and another carries S29/V92. However, they are not listed in the table here.

V_L_ S29R in a IGHV1-58/IGKV3-20 public clonotype represented a previously unknown recurring SHM (Figure 4B). V_L_ S29R emerged in 8 out of 26 (31%) donors that carried this IGHV1-58/IGKV3-20 public clonotype. Antibodies of this IGHV1-58/IGKV3-20 public clonotype bind to the ridge region of SARS-CoV-2 RBD (Figure 5A) and are able to potently neutralize multiple variants of concern (VOCs) (Li et al., 2021b; Schmitz et al., 2021; Wang et al., 2021), including Omicron (Zhou et al., 2022a). Furthermore, therapeutic antibody tixagevimab is derived from a member of this IGHV1-58/IGKV3-20 public clonotype, namely COV2-2196 (Dong et al., 2021). Here, we compared two previously determined structures of IGHV1-58/IGKV3-20 antibodies in complex with RBD (Dejnirattisai et al., 2021; Wheatley et al., 2021). One has the germline-encoded V_L_ S29 (Figure 5B) and the other carries a somatically mutated V_L_ R29 (Figure 5C). While neither V_L_ S29 nor V_L_ R29 directly interact with RBD, V_L_ R29 is able to form a cation-π interaction with V_L_ Y32, which in turn forms a T-shaped π-π stacking with RBD-F486 and H-bonds with RBD-C480 (Figure 5C). The positioning of V_L_ R29 can further be stabilized by a salt bridge with another SHM V_L_ G92D (Figure 5C). The RBD binding affinity of COVOX-253, which is an IGHV1-58/IGKV3-20-encoded antibody, was improved >3-fold by the V_L_ S29R/G92D double mutant but only subtly enhanced or diminished by V_L_ S29R or V_L_ G92D, respectively (Figure 5D), indicating a synergistic effect between V_L_ S29R and V_L_ G92D. In fact, V_L_ G92D seemed to have coevolved with V_L_ S29R, since V_L_ G92D was found in four out of the 67 antibodies in this IGHV1-58/IGKV3-20 public clonotype and all four carried V_L_ S29R (Figure 5E). Moreover, a phylogenetic analysis showed that V_L_ G92D emerged from a cluster of antibodies with V_L_ S29R (Figure 5E). These analyses illustrate that recurring SHMs are associated with the public antibody response to SARS-CoV-2 S and further suggest the existence of common affinity maturation pathways that involve emergence of multiple SHMs in a defined order.

### Deep learning enables classification of antibody specificity

Since many sequence features of public antibody responses to the S protein could be observed in our dataset, we postulated that the dataset was sufficiently large to train a deep learning model to identify S antibodies. To provide a proof of concept, we trained a deep learning model to distinguish between human antibodies to S and to influenza HA. Among different antigens, HA was chosen here because there were a large number of HA antibodies with published sequences, albeit still lower than the published SARS-CoV-2 S antibodies. Here, 1,356 unique human antibodies to HA and 3,000 unique human antibodies to SARS-CoV-2 S with complete information for all six CDR sequences were used (Table S3). None of these antibodies had identical sequences in all six CDRs. These antibodies to S and HA were divided into a training set (64%), a validation set (16%), and a test set (20%), with no overlap between the three sets. The overlap of clonotypes was also minimal (Figure S6A). Subsequently, the training set was used to train the deep learning model. The validation set was used to evaluate the model performance during training. The test set was used to evaluate the performance of the final model.

**Figure 6:**
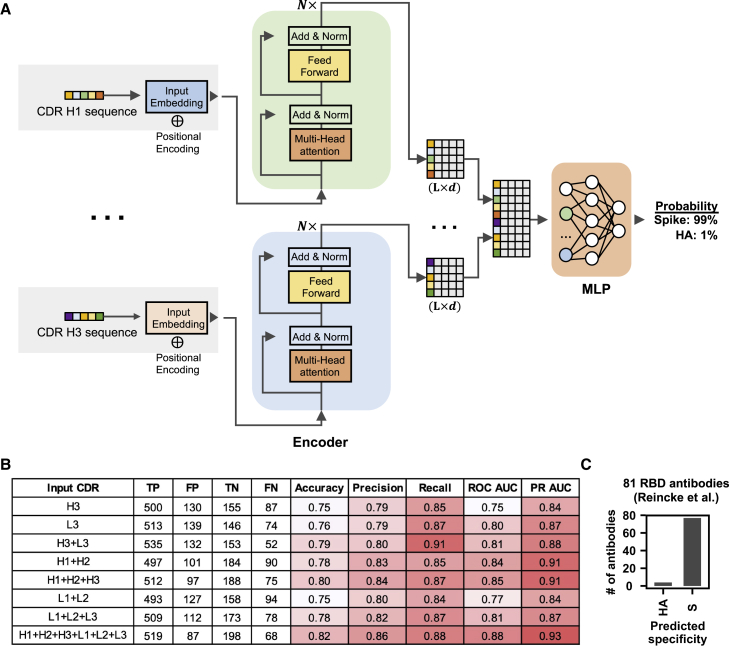
Specificity of antibodies can be predicted by a sequence-based deep learning model (A) A schematic overview of the deep learning model architecture. (B) For evaluating model performance, S antibodies and HA antibodies were considered “positive” and “negative,” respectively. Model performance on the test set was compared when different input types were used. Of note, the test set has no overlap with the training set and the validation set, both of which were used to construct the deep learning model. True positive (TP) represents the number of S antibodies being correctly classified as S antibodies. False positive (FP) represents the number of HA antibodies being misclassified as S antibodies. True negative (TN) represents the number of HA antibodies being correctly classified as HA antibodies. False negative (FN) represents the number of S antibodies being misclassified as HA antibodies. See STAR Methods for the calculations of accuracy, precision, recall, ROC AUC, and PR AUC. (C) The antigen specificity of 81 RBD antibodies from Reincke et al. (2022) were predicted by a deep learning model that was trained to distinguish between S antibodies and HA antibodies. See also Figure S6 and Table S3. The dataset for constructing and testing the deep-learning model, related to Figure 6, Table S4. Performance of the deep learning model with different inputs, related to Figure 6, Table S5. Prediction result of 81 antibodies to SARS-CoV-2 RBD that were elicited by Beta variant infection, related to Figure 6, Table S6. Prediction result of 691 HIV antibodies from GenBank, related to Figure 6.

Our deep learning model had a simple architecture, which consisted of one encoder per CDR followed by three fully connected layers (Figure 6A). To evaluate the model performance on the test set, the area under the curves of receiver operating characteristic (ROC AUC) and precision-recall (PR AUC) were used to measure the model's ability to avoid misclassification (Flach et al., 2011; Saito and Rehmsmeier, 2015). Model performance was the best when all six CDRs (i.e., H1, H2, H3, L1, L2, and L3) were used to train the model, which resulted in an ROC AUC and an PR AUC of 0.88 and 0.93, respectively (Figure 6B; Table S4). However, reasonable performance was also observed when the model was trained by a subset of CDRs (AUCs = 0.75–0.85 and PR AUCs = 0.84–0.91). These results are consistent with the notion that the public antibody response to SARS-CoV-2 is composed of diverse sequence features on both heavy and light chains.

We further tested if a deep learning model could be trained to distinguish antibodies to different domains of S, namely RBD, NTD, and S2. Since the numbers of NTD and S2 antibodies were small, the model was trained by the heavy-chain CDRs (H1, H2, and H3), so that antibodies without sequence information for the light chain could also be used (Table S3). The ROC AUC and PR AUC of the RBD/NTD/S2 model were 0.79 and 0.62, respectively (Figure S6B), which were much worse than the S/HA model above. The poorer performance of the RBD/NTD/S2 model may be attributable to the smaller dataset. Since most known antibodies to SARS-CoV-2 S were RBD-specific, we also examined if a deep learning model that was trained to distinguish RBD and HA antibodies could achieve a better performance than the S/HA model above. Indeed, the ROC AUC and PR AUC of the RBD/HA model were 0.90 and 0.94, respectively (Figure S6B; Table S3), which were slightly higher than those of the S/HA model. These observations indicate that the size of the training dataset is indeed critical for model performance.

A recent study reported 81 antibodies to SARS-CoV-2 RBD that were elicited by Beta variant infection, in which 44 could cross-react with the ancestral Hu-1 strain and 37 were Beta-specific (Reincke et al., 2022). While these 81 antibodies were not included in the dataset that we assembled (Table S1), they provided an opportunity to further evaluate the performance of our deep learning model. Our deep learning model that was trained on all six CDRs to distinguish between antibodies to S and HA (see above) successfully predicted that 77 of the 81 (95%) antibodies as SARS-CoV-2 S antibodies (Figure 6C; Table S5). Of note, since our model was designed to distinguish between antibodies to SARS-CoV-2 S and influenza HA, the prediction on non-S/non-HA antibodies was expected to be close to random. Consistent with that expectation, when we applied our model to 691 HIV antibodies from GenBank (Table S6), 46% were predicted to be S antibodies and 54% were predicted to be HA antibodies (Figure S6C). As different antigenic variants of SARS-CoV-2 emerge and individuals start to accumulate unique SARS-CoV-2 immune histories, the antibody response to SARS-CoV-2 is likely to evolve and diversify. Although our model still performs well on antibodies that were elicited by the Beta variant (Figure 6C), it remains to be explored whether this performance will hold for antibodies that are elicited by SARS-CoV-2 variants that are more antigenically distinct from the ancestral Hu-1 strain originally identified in Wuhan.

## Discussion

Through a systematic survey of published information on SARS-CoV-2 antibodies, we identified many molecular features of public antibody responses to SARS-CoV-2. The large amount of published information has allowed us to explore distinct patterns of germline gene usages in antibodies that target different domains on the S protein (i.e., RBD, NTD, and S2). Notably, the types and nature of public antibody responses to different domains appear to be quite different. For example, convergence of CDR H3 sequences can be readily identified in the public antibody responses to RBD and S2. In contrast, the public antibody response to NTD seems to be largely independent of the CDR H3 sequence. Furthermore, an IGHD-dependent public antibody response was enriched against S2, but not RBD or NTD. Together, our study demonstrates the diversity of sequence features that can constitute a public antibody response against a single antigen.

The public antibody response to SARS-CoV-2 has also been examined by a recent data mining study that focused on identifying public clonotypes (Chen et al., 2021). This previous study defined public clonotypes as antibodies with the same IGHV/IGHJ/IGK(L)V/IGK(L)V genes and high similarity of CDR H3 (Chen et al., 2021). While multiple public clonotypes were identified using this stringent definition (Chen et al., 2021), the characterization of public antibody response is likely far from complete. A public antibody response may not always involve a defined pair of IGHV/IGK(L)V genes, especially when either IGHV or IGK(L)V gene-encoded residues only make a minimal contribution to the paratope. In fact, a well-characterized public antibody response to the highly conserved stem region of influenza HA has a paratope that is entirely attributed to the IGHV1-69 heavy chain (Dreyfus et al., 2012; Ekiert et al., 2009; Lang et al., 2017; Sui et al., 2009). IGHV3-30/IGHD1-26 antibodies to S2 in our study may represent a similar type of IGK(L)V-independent public antibody response, although it still needs to be confirmed by structural analysis. On the other extreme, RBD antibodies that are encoded by IGLV6-57 with a ^97^WLRG^100^ motif in the CDR H3 represent a public response that is largely independent of IGHV gene usage. Given the diverse types of public antibody responses to SARS-CoV-2 S, we need to acknowledge the limitation of using the conventional strict definition of public clonotype to study public antibody responses.

Public antibody response to different antigens can have very different sequence features. For example, IGHV6-1 and IGHD3-9 are signatures of public antibody response to influenza virus (Joyce et al., 2016; Kallewaard et al., 2016; Wu et al., 2018, 2020a), whereas IGHV3-23 is frequently used in antibodies to Dengue and Zika viruses (Robbiani et al., 2017). In contrast, these germline genes are seldom used in the antibody response to SARS-CoV-2 as compared with the naive baseline. Since the binding specificity of an antibody is determined by its structure, which in turn is determined by its amino acid sequence, the antigen specificity of an antibody can theoretically be identified based on its sequence. This study provides a proof of concept by training a deep learning model to distinguish between SARS-CoV-2 S antibodies and influenza HA antibodies, solely based on primary sequence information. Technological advancements, such as the development of single-cell high-throughput screen using the Berkeley Lights Beacon optofluidics device (Winters et al., 2019) and advances in paired B cell receptor sequencing (Curtis and Lee, 2020), have been accelerating the speed of antibody discovery and characterization. As more sequence information on antibodies to different antigens is accumulated, we may be able in the future to construct a generalized sequence-based model to accurately predict the antigen specificity of any antibody.

In summary, the amount of publicly available information on SARS-CoV-2 antibodies has provided invaluable biological insights that have not been readily obtained for other pathogens. One reason is that the COVID-19 pandemic has gathered scientists from many fields and around the globe to work intensively on SARS-CoV-2. The parallel efforts by many different research groups have enabled SARS-CoV-2 antibodies to be discovered at unprecedented speed and scale that have not been possible for other pathogens. We anticipate that knowledge of the molecular features of the antibody response to SARS-CoV-2 will keep growing as more antibodies are isolated and characterized. Ultimately, the extensive characterization of antibodies to the SARS-CoV-2 S protein may allow us to address some of the most fundamental questions about antigenicity and immunogenicity, as well as how the human immune repertoire has evolved to respond to specific classes of viral pathogens that have coexisted with humans for hundreds to thousands of years.

### Limitations of the study

Many antibodies in our collection were isolated from SARS-CoV-2-infected individuals. However, sequence information of the infecting viral variants was not available in the original publications. Although most of these antibodies were isolated during the early phase of the COVID-19 pandemic, some antibodies in our collection may have been elicited by a SARS-CoV-2 variant rather than the ancestral Hu-1 strain. Relatedly, this study did not examine the antibody specificity to different variants. By leveraging the published information on antibody neutralization activity to different variants, future analysis could investigate the relationship between antibody sequence features and neutralization breadth.

## STAR★Methods

### Key resources table

**Table:** 

REAGENT or RESOURCE	SOURCE	IDENTIFIER
ExpiCHO Expression System Kit	Thermo Fisher Scientific	Cat# A29133
Expi293 Expression System Kit	ThermoFisher	Cat# A14635
Phosphate-buffered saline (PBS)	Thermo Fisher Scientific	Cat# 14040133
Ni Sepharose excel resin	Cytiva	Cat# 17371202
CaptureSelect CH1-XL Affinity Matrix	Thermo Fisher Scientific	Cat# 1943462010

**Chemicals and recombinant proteins**		

Sodium chloride (NaCl)	Sigma-Aldrich	Cat# S9888
Concentrated hydrochloric acid (HCl)	Sigma-Aldrich	Cat# H1758
Bovine Serum Albumin (BSA)	Sigma-Aldrich	Cat# A9418
Tween 20	Fisher Scientific	Cat# BP337-500

**Critical commercial assays**		

In-Fusion HD Cloning Kit	Takara	Cat# 639647
KOD Hot Start DNA Polymerase	EMD Millipore	Cat# 71086-3
PCR Clean-Up and Gel Extraction Kit	Clontech Laboratories	Cat# 740609.250
QIAprep Spin Miniprep Kit	Qiagen	Cat# 27106
NucleoBond Xtra Maxi	Clontech Laboratories	Cat# 740414.100

**Deposited data**		

Collection of antibody information	This study	Table S1
Custom scripts	This study	https://doi.org/10.5281/zenodo.6370701

**Cell lines**		

ExpiCHO cells	Thermo Fisher Scientific	Cat# A29127; RRID:CVCL_5J31
Expi293F cells	Thermo Fisher Scientific	Cat# A14527; RRID:CVCL_D615

**Recombinant DNA**		

phCMV3-COVOX-253 Fab heavy chain	This study	N/A
phCMV3-COVOX-253 Fab light chain	This study	N/A
phCMV3-SARS-CoV-2-RBD	(Wu et al., 2020b)	N/A

**Software and algorithms**		

Octet analysis software 9.0	Fortebio	N/A
Python	https://www.python.org/	N/A
R	https://www.r-project.org/	N/A
IgBLAST	(Ye et al., 2013)	N/A
Logomaker	(Tareen and Kinney, 2020)	N/A
ANARCI	(Dunbar and Deane, 2016)	N/A
MAFFT	(Katoh and Standley, 2013)	N/A
FastTree	(Price et al., 2010)	N/A
ggtree	(Yu, 2020)	N/A
PyIR	(Soto et al., 2020)	N/A
TensorFlow	(Abadi et al., 2016)	N/A

**Other**		

Fab-CH1 2nd generation (FAB2G) biosensors	ForteBio	Cat# 18-5019

### Resource availability

#### Lead contact

Further information and requests for resources and reagents should be directed to and will be fulfilled by the lead contact, by the lead contact, Nicholas C. Wu (nicwu@illinois.edu).

### Experimental model and subject details

#### Cell cultures

ExpiCHO cells (Chinese hamster ovary cells, female) and Expi293F cells (human embryonic kidney cells, female) were maintained in ExpiCHO expression medium and Expi293 expression medium, respectively, at 37°C with 8% CO_2_ according to the manufacturer’s instructions (Thermo Fisher Scientific).

### Method details

#### Collection of antibody information

Information on the monoclonal antibodies is derived from the original papers (Table S1). Sequences of each monoclonal antibody are from the original papers and/or NCBI GenBank database (www.ncbi.nlm.nih.gov/genbank) (Benson et al., 2013). Putative germline genes were identified by IgBLAST (Ye et al., 2013). Some studies isolated antibodies from multiple donors, but the donor identity for each antibody was not always clear. For example, some studies mixed B cells from multiple donors before isolating individual B cell clones. Since the donor identity cannot be distinguished among those antibodies, we considered them from the same donor with “_mix” as the suffix of the donor ID. In addition, the PBMCs of SARS-CoV survivors in three separate studies were all from NIH/VRC (Li et al., 2021a; Shiakolas et al., 2021; Wec et al., 2020). Since it is unclear If they are the same SARS-CoV survivor, the same donor ID “VRC_SARS1” was assigned to them to avoid overestimation of public antibody response. If the neutralization activity of a given antibody was only measured at a single concentration, 50% neutralization activity or below was classified as non-neutralizing. We also downloaded the CoV-AbDab (Raybould et al., 2021) in September 2021 to fill in any additional information. As of September 2021, there were 2,582 human SARS-CoV-2 antibodies in CoV-AbDab. Information in the finalized dataset was manually inspected by three different individuals. For antibodies that were shown to bind to S1 but not RBD, they were classified as NTD antibodies. Due to having identical nucleotide sequences, IGKV1D-39^∗^01 was classified as IGKV1-39^∗^01, IGHV1-68D^∗^02 as IGHV1-68^∗^02, IGHV1-69D^∗^01 as IGHV1-69^∗^19, IGHV3-23D^∗^01 as IGHV3-23^∗^01, and IGHV3-29^∗^01 as IGHV3-30-42^∗^01.

#### Analysis of germline gene usages

Non-functional germline genes were ignored in our germline gene usage analysis. Except for the analysis presented in Figure 1, IGHV3-30-3 was classified as IGHV3-30 since they have identical amino-acid sequence in the framework regions, CDR H1, and CDR H2. To establish the baseline germline usage frequency, published antibody repertoire sequencing datasets from 26 healthy donors (Briney et al., 2019; Soto et al., 2019) were downloaded from cAb-Rep (Guo et al., 2019). Putative germline genes for each antibody sequence in these repertoire sequencing datasets from healthy donors were identified by IgBLAST (Ye et al., 2013).

#### CDR H3 clustering analysis

Using a deterministic clustering approach, antibodies with CDR H3 sequences that had the same length and at least 80% amino-acid sequence identity were assigned to the same cluster. As a result, CDR H3 of every antibody in a cluster would have >20% difference in amino-acid sequence identity with that of every antibody in another cluster. A cluster would be discarded if all of its antibody members were from the same donor. The number of antibodies within a cluster was defined as the cluster size. Sequence logos were generated by Logomaker in Python (Tareen and Kinney, 2020). For each cluster, epitope assignment was performed using the following scoring scheme. Briefly, there were three scoring categories, namely “RBD”, “NTD”, and “S2”.

• 1 point was added to category “RBD” for each antibody with an epitope label equals to “S:RBD” or “S:S1”.
• 1 point was added to category “NTD” for each antibody with an epitope label equals to “S:NTD”, “S:S1”, “S:non-RBD”, or “S:S1 non-RBD”.
• 1 point was added to category “S2” for each antibody with an epitope label equals to “S:S2”, ” S:S2 Stem Helix”, “S:non-RBD”.

The category with >50% of the total points would be classified as the epitope for a given cluster. If no category had >50% of the total points, the epitope for the cluster would be classified as “unknown”.

#### Identification of recurring somatic hypermutation (SHM)

In this analysis, a public clonotype was classified as antibodies from at least two donors that had the same IGHV/IGK(L)V genes and CDR H3s from the same CDR H3 cluster (see “CDR H3 clustering analysis” above). For each antibody, ANARCI was used to number the position of each residue according to Kabat numbering (Dunbar and Deane, 2016). The amino-acid identity at each residue position of an antibody was then compared to that of the putative germline gene. CDR H3, CDR L3, and framework region 4 in both heavy and light chains were not included in this analysis. Insertions and deletions were also ignored in this analysis. SHM that occurred in at least two donors within a public clonotype was defined as a recurring SHM.

#### Expression and purification of SARS-CoV-2 RBD

SARS-CoV-2 spike receptor-binding domain (RBD) was expressed in mammalian cells and purified as described previously (Wu et al., 2020b). Briefly, the RBD (residues 319-541) of the SARS-CoV-2 spike (S) protein (GenBank: QHD43416.1) was cloned into a phCMV3 vector and fused with a C-terminal 6xHis tag. The plasmid was transiently transfected into Expi293F cells using ExpiFectamine 293 Reagent (Thermo Fisher Scientific) according to the manufacturer’s instructions. The supernatant was collected at 7 days post-transfection. The protein was purified with Ni Sepharose excel resin (Cytiva) followed by size exclusion chromatography.

#### Expression and purification of Fabs

The heavy and light chains were individually cloned into a phCMV3 vector. The plasmids were transiently co-transfected into ExpiCHO cells at a ratio of 2:1 (HC:LC) using ExpiFectamine CHO Reagent (Thermo Fisher Scientific) according to the manufacturer’s instructions. The supernatant was collected at 7 days post-transfection. The Fabs were purified with a CaptureSelect CH1-XL Affinity Matrix (Thermo Fisher Scientific) followed by size exclusion chromatography.

#### Biolayer interferometry binding assay

Binding assays were performed by biolayer interferometry (BLI) using an Octet Red instrument (FortéBio). To measure the binding kinetics of anti-SARS-CoV-2 Fabs and RBD, Fabs were diluted with kinetic buffer (1x PBS, pH 7.4, 0.01% BSA and 0.002% Tween 20) into 50 μg/ml, then loaded onto Octet FAB2G biosensors and interacted with SARS-CoV-2 RBDs. Binding kinetics were measured for five concentrations of RBDs at 3-fold dilution ranging from 300 nM to 3.7 nM. The assay consisted of the following steps. 1) baseline: 1 min with 1x kinetic buffer; 2) loading: 120 seconds with Fabs; 3) wash: 30 seconds wash of unbound Fabs with 1x kinetic buffer; 4) baseline: 1 min with 1x kinetic buffer; 5) association: 90 seconds with RBDs; and 6) dissociation: 90 seconds with 1x kinetic buffer. For estimating the dissociation constant (*K*_D_), a 1:1 binding model was used.

#### Phylogenetic tree construction

Amino acid residues 1-94 (Kabat numbering) in the light chain sequences of IGHV1-58/IGKV3-20 antibodies from CDR H3 cluster 3 were aligned using MAFFT (Katoh and Standley, 2013). The phylogenetic tree was generated using FastTree (Price et al., 2010) and visualized using ggtree (Yu, 2020).

#### Ramachandran plot

Ramachandran plots were generated using the Ramachandran Plot Server (https://zlab.umassmed.edu/bu/rama/) (Anderson et al., 2005).

#### Deep learning model for antigen identification

##### Model construction

The deep learning model consisted of two networks, namely multi-encoder (ME) and a stack of multi-layered perceptrons (MLP). The CDR amino-acid sequences were taken as input and passed to ME. Specifically, each CDR amino-acid sequence was described by a 21-letter alphabet vector $\vec{x}=\left(x_{1},x_{2},\ldots ,x_{L-1},x_{L}\right),x\;\in \;R^{L}$, where L represented the length of sequence, and x represented the amino acid category. Each of the 20 canonical amino acids was one category, whereas all the ambiguous amino acids were grouped as the 21^st^ category. Before passing to ME, tokenized amino acid sequences were processed by zero padding, so that the size of each input was the same. Subsequently, the inputs were mapped to the embedding vectors with additional dimension d. The sinusoidal positional encoding vectors were added to the embedding vectors to encode the relative position of tokens (i.e. amino acids) in the sequence. Each embedding vector, $\vec{x}\;\in \;R^{L\times d}$, with size of L×d, was passed into transformer encoder layer by self-attention mechanism to learn the sequence feature (Vaswani et al., 2017). All learned sequence features were then concatenated together and passed to multi-layered perceptron (MLP). Each MLP layer contained leaky rectified linear unit (ReLU) activations to avoid the vanishing gradient. Dropout layers were placed after each MLP block to avoid model overfitting (Srivastava et al., 2014). The final output layer was followed by a sigmoid activation function to predict the probability of different classes. The prediction losses were calculated by binary cross-entropy loss.

##### Training detail

SARS-CoV-2 S antibodies and influenza HA antibodies with complete information for all six CDR sequences were identified. Sequences of each HA antibody were from NCBI GenBank database (www.ncbi.nlm.nih.gov/genbank) (Benson et al., 2013) (Table S3). If all six CDR sequences were the same between two or more antibodies, only one of these antibodies would be retained. After filtering duplicates, there were 4,736 antibodies to SARS-CoV-2 and 1,356 to influenza HA. To avoid data imbalance, we further down-sampled to 3,000 SARS-CoV-2 antibodies. The CDR sequences were identified by IgBLAST and PyIR (Soto et al., 2020; Ye et al., 2013). This dataset was randomly split into a training set (64%), a validation set (16%), and a test set (20%). The training set was used to train the deep learning model. The validation set was used to evaluate the model performance during training. The test set was used to evaluate the performance of the final model. There was no overlap of antibody sequences among the training set, validation set, and test set. The Adam algorithm was used to optimize the model. The following hyper-parameters were used for model training:

CDR embedding size: 256

The number of attention heads for self-attention on CDR feature learning: 4

The number of encoder layer for CDR encoder: 4

Size of stacking MLP layers: 512, 128, and 64

Learning rate: 0.0001

Batch size: 256

Using the same training set, validation set and test set, the model performance of using the following inputs was compared:

CDR H1 + H2

CDR L1 + L2

CDR H3

CDR L3

CDR H3 + L3

CDR H1 + H2 + H3

CDR L1 + L2 + L3

CDR H1 + H2 + H3 + L1 + L2 + L3

The same procedure was used for training the RBD/NTD/S2 model or the RBD/HA model, except that the prediction losses for RBD/NTD/S2 model were calculated by categorical cross-entropy loss since it has more than two categories. For the RBD/NTD/S2 model, the number of RBD antibodies were down-sampled to 800. Without down-sampling the RBD antibodies, the model would be highly biased towards RBD, with very low recall rates of 0.39 and 0.16 for S2 antibodies and NTD antibodies, respectively. For the RBD/HA model, 3000 RBD antibodies and 1,356 HA antibodies were used.

##### Performance Metrics

For evaluating model performance, S antibodies and HA antibodies were considered “positive” and “negative”, respectively. False positives (FP) and false negatives (FN) were samples that were misclassified by the model while true negatives (TN) and true positives (TP) were correctly classified ones. The following metrics were computed to evaluate model performance:

(Equation 1) $$accuarcy=\frac{TP+TN}{TP+FN+FP+TN}$$

(Equation 2) $$precision=\frac{TP}{TP+FP}$$

(Equation 3) $$recall=\frac{TP}{TP+FN}$$

In addition, we also used the receiver operating characteristic (ROC) curve and precision-recall (PR) curve to measure the model's ability to avoid misclassification (Flach et al., 2011; Saito and Rehmsmeier, 2015). Area under the curves of ROC (i.e. ROC AUC) and PR (i.e. PR AUC) were computed using the "keras.metrics" module in TensorFlow (Abadi et al., 2016).

### Quantification and statistical analysis

Standard deviation for K_D_ estimation was computed by Octet analysis software 9.0.

## Data Availability

• The assembled SARS-CoV-2 antibody dataset is in Table S1. The dataset for constructing and testing the deep learning model is in Table S3. Additional Supplemental Items are available from Mendeley Data at http://doi.org/10.17632/wzdvt6g3cz.1. • Custom python scripts for all analyses have been deposited to Zenodo at https://doi.org/10.5281/zenodo.6370701. • Any additional information required to reanalyze the data reported in this paper is available from the lead contact upon request.

## Supplemental information

Document S1. Figures S1–S6

Table S1. Collection of SARS-CoV-2 antibody information, related to Figures 1, 2, 3, and 4

Table S2. Neutralization activity and binding affinity of antibodies in CDR H3 clusters 5, 10, and 15; RBD antibodies encoded by IGHV3-13/IGKV1-39; and IGHD1-26 S2 antibodies, related to Figures 1, 2, and 3

Table S3. The dataset for constructing and testing the deep-learning model, related to Figure 6

Table S4. Performance of the deep learning model with different inputs, related to Figure 6

Table S5. Prediction result of 81 antibodies to SARS-CoV-2 RBD that were elicited by Beta variant infection, related to Figure 6

Table S6. Prediction result of 691 HIV antibodies from GenBank, related to Figure 6

Document S2. Article plus supplemental information

## References

[bib1] Abadi M., Barham P., Chen J., Chen Z., Davis A., Dean J., Devin M., Ghemawat S., Irving G., Isard M. (2016).

[bib2] Álvarez-Prado Á.F., Pérez-Durán P., Pérez-García A., Benguria A., Torroja C., de Yébenes V.G., Ramiro A.R. (2018). A broad atlas of somatic hypermutation allows prediction of activation-induced deaminase targets. J. Exp. Med..

[bib3] Anderson R.J., Weng Z., Campbell R.K., Jiang X. (2005). Main-chain conformational tendencies of amino acids. Proteins.

[bib4] Andrews S.F., McDermott A.B. (2018). Shaping a universally broad antibody response to influenza amidst a variable immunoglobulin landscape. Curr. Opin. Immunol..

[bib5] Barnes C.O., Jette C.A., Abernathy M.E., Dam K.A., Esswein S.R., Gristick H.B., Malyutin A.G., Sharaf N.G., Huey-Tubman K.E., Lee Y.E. (2020). SARS-CoV-2 neutralizing antibody structures inform therapeutic strategies. Nature.

[bib6] Benson D.A., Cavanaugh M., Clark K., Karsch-Mizrachi I., Lipman D.J., Ostell J., Sayers E.W. (2013). GenBank. Nucleic Acids Res..

[bib7] Briney B., Inderbitzin A., Joyce C., Burton D.R. (2019). Commonality despite exceptional diversity in the baseline human antibody repertoire. Nature.

[bib8] Cao Y., Su B., Guo X., Sun W., Deng Y., Bao L., Zhu Q., Zhang X., Zheng Y., Geng C. (2020). Potent neutralizing antibodies against SARS-CoV-2 identified by high-throughput single-cell sequencing of convalescent patients' B cells. Cell.

[bib9] Cerutti G., Guo Y., Zhou T., Gorman J., Lee M., Rapp M., Reddem E.R., Yu J., Bahna F., Bimela J. (2021). Potent SARS-CoV-2 neutralizing antibodies directed against spike N-terminal domain target a single Supersite. Cell Host Microbe.

[bib10] Chen E.C., Gilchuk P., Zost S.J., Suryadevara N., Winkler E.S., Cabel C.R., Binshtein E., Chen R.E., Sutton R.E., Rodriguez J. (2021). Convergent antibody responses to the SARS-CoV-2 spike protein in convalescent and vaccinated individuals. Cell Rep..

[bib11] Chi X., Yan R., Zhang J., Zhang G., Zhang Y., Hao M., Zhang Z., Fan P., Dong Y., Yang Y. (2020). A neutralizing human antibody binds to the N-terminal domain of the Spike protein of SARS-CoV-2. Science.

[bib12] Clark S.A., Clark L.E., Pan J., Coscia A., McKay L.G.A., Shankar S., Johnson R.I., Brusic V., Choudhary M.C., Regan J. (2021). SARS-CoV-2 evolution in an immunocompromised host reveals shared neutralization escape mechanisms. Cell.

[bib13] Curtis N.C., Lee J. (2020). Beyond bulk single-chain sequencing: getting at the whole receptor. Curr. Opin. Syst. Biol..

[bib14] Dejnirattisai W., Zhou D., Ginn H.M., Duyvesteyn H.M.E., Supasa P., Case J.B., Zhao Y., Walter T.S., Mentzer A.J., Liu C. (2021). The antigenic anatomy of SARS-CoV-2 receptor binding domain. Cell.

[bib15] Di Noia J.M., Neuberger M.S. (2007). Molecular mechanisms of antibody somatic hypermutation. Annu. Rev. Biochem..

[bib16] Dong J., Zost S.J., Greaney A.J., Starr T.N., Dingens A.S., Chen E.C., Chen R.E., Case J.B., Sutton R.E., Gilchuk P. (2021). Genetic and structural basis for SARS-CoV-2 variant neutralization by a two-antibody cocktail. Nat. Microbiol..

[bib17] Dreyfus C., Laursen N.S., Kwaks T., Zuijdgeest D., Khayat R., Ekiert D.C., Lee J.H., Metlagel Z., Bujny M.V., Jongeneelen M. (2012). Highly conserved protective epitopes on influenza B viruses. Science.

[bib18] Dunbar J., Deane C.M. (2016). ANARCI: antigen receptor numbering and receptor classification. Bioinformatics.

[bib19] Ekiert D.C., Bhabha G., Elsliger M.A., Friesen R.H., Jongeneelen M., Throsby M., Goudsmit J., Wilson I.A. (2009). Antibody recognition of a highly conserved influenza virus epitope. Science.

[bib20] Elhanati Y., Sethna Z., Marcou Q., Callan C.G., Mora T., Walczak A.M. (2015). Inferring processes underlying B-cell repertoire diversity. Philos. Trans. R. Soc. Lond. B Biol. Sci..

[bib21] Flach P., Hernández-Orallo J., Ferri C. (2011). Proceedings of the 28th international conference on International Conference on Machine Learning.

[bib22] Graham C., Seow J., Huettner I., Khan H., Kouphou N., Acors S., Winstone H., Pickering S., Galao R.P., Dupont L. (2021). Neutralization potency of monoclonal antibodies recognizing dominant and subdominant epitopes on SARS-CoV-2 Spike is impacted by the B.1.1.7 variant. Immunity.

[bib23] Guo Y., Chen K., Kwong P.D., Shapiro L., Sheng Z. (2019). cAb-Rep: a database of curated antibody repertoires for exploring antibody diversity and predicting antibody prevalence. Front. Immunol..

[bib24] Henry Dunand C.J., Wilson P.C. (2015). Restricted, canonical, stereotyped and convergent immunoglobulin responses. Philos. Trans. R. Soc. Lond. B Biol. Sci..

[bib25] Hurlburt N.K., Seydoux E., Wan Y.H., Edara V.V., Stuart A.B., Feng J., Suthar M.S., McGuire A.T., Stamatatos L., Pancera M. (2020). Structural basis for potent neutralization of SARS-CoV-2 and role of antibody affinity maturation. Nat. Commun..

[bib26] Jackson K.J., Liu Y., Roskin K.M., Glanville J., Hoh R.A., Seo K., Marshall E.L., Gurley T.C., Moody M.A., Haynes B.F. (2014). Human responses to influenza vaccination show seroconversion signatures and convergent antibody rearrangements. Cell Host Microbe.

[bib27] Joyce M.G., Wheatley A.K., Thomas P.V., Chuang G.Y., Soto C., Bailer R.T., Druz A., Georgiev I.S., Gillespie R.A., Kanekiyo M. (2016). Vaccine-induced antibodies that neutralize group 1 and group 2 influenza A viruses. Cell.

[bib28] Jung D., Alt F.W. (2004). Unraveling V(D)J recombination; insights into gene regulation. Cell.

[bib29] Kallewaard N.L., Corti D., Collins P.J., Neu U., McAuliffe J.M., Benjamin E., Wachter-Rosati L., Palmer-Hill F.J., Yuan A.Q., Walker P.A. (2016). Structure and function analysis of an antibody recognizing all influenza A subtypes. Cell.

[bib30] Katoh K., Standley D.M. (2013). MAFFT multiple sequence alignment software version 7: improvements in performance and usability. Mol. Biol. Evol..

[bib31] Kim S.I., Noh J., Kim S., Choi Y., Yoo D.K., Lee Y., Lee H., Jung J., Kang C.K., Song K.H. (2021). Stereotypic neutralizing VH antibodies against SARS-CoV-2 spike protein receptor binding domain in patients with COVID-19 and healthy individuals. Sci. Transl. Med..

[bib32] Krissinel E., Henrick K. (2007). Inference of macromolecular assemblies from crystalline state. J. Mol. Biol..

[bib33] Lang S., Xie J., Zhu X., Wu N.C., Lerner R.A., Wilson I.A. (2017). Antibody 27F3 broadly targets influenza A group 1 and 2 hemagglutinins through a further variation in VH1-69 antibody orientation on the HA stem. Cell Rep..

[bib34] Lanzavecchia A., Frühwirth A., Perez L., Corti D. (2016). Antibody-guided vaccine design: identification of protective epitopes. Curr. Opin. Immunol..

[bib35] Li D., Edwards R.J., Manne K., Martinez D.R., Schäfer A., Alam S.M., Wiehe K., Lu X., Parks R., Sutherland L.L. (2021). *In vitro* and *in vivo* functions of SARS-CoV-2 infection-enhancing and neutralizing antibodies. Cell.

[bib36] Li D., Sempowski G.D., Saunders K.O., Acharya P., Haynes B.F. (2022). SARS-CoV-2 neutralizing antibodies for COVID-19 prevention and treatment. Annu. Rev. Med..

[bib37] Li T., Han X., Gu C., Guo H., Zhang H., Wang Y., Hu C., Wang K., Liu F., Luo F. (2021). Potent SARS-CoV-2 neutralizing antibodies with protective efficacy against newly emerged mutational variants. Nat. Commun..

[bib38] Li W., Chen Y., Prévost J., Ullah I., Lu M., Gong S.Y., Tauzin A., Gasser R., Vézina D., Anand S.P. (2022). Structural basis and mode of action for two broadly neutralizing antibodies against SARS-CoV-2 emerging variants of concern. Cell Rep..

[bib39] Piccoli L., Park Y.J., Tortorici M.A., Czudnochowski N., Walls A.C., Beltramello M., Silacci-Fregni C., Pinto D., Rosen L.E., Bowen J.E. (2020). Mapping neutralizing and immunodominant sites on the SARS-CoV-2 spike receptor-binding domain by structure-guided high-resolution serology. Cell.

[bib40] Pieper K., Tan J., Piccoli L., Foglierini M., Barbieri S., Chen Y., Silacci-Fregni C., Wolf T., Jarrossay D., Anderle M. (2017). Public antibodies to malaria antigens generated by two LAIR1 insertion modalities. Nature.

[bib41] Pinto D., Sauer M.M., Czudnochowski N., Low J.S., Tortorici M.A., Housley M.P., Noack J., Walls A.C., Bowen J.E., Guarino B. (2021). Broad Betacoronavirus neutralization by a stem helix-specific human antibody. Science.

[bib42] Price M.N., Dehal P.S., Arkin A.P. (2010). FastTree 2--approximately maximum-likelihood trees for large alignments. PLoS One.

[bib43] Raybould M.I.J., Kovaltsuk A., Marks C., Deane C.M. (2021). CoV-AbDab: the coronavirus antibody database. Bioinformatics.

[bib44] Reincke S.M., Yuan M., Kornau H.C., Corman V.M., van Hoof S., Sánchez-Sendin E., Ramberger M., Yu W., Hua Y., Tien H. (2022). SARS-CoV-2 Beta variant infection elicits potent lineage-specific and cross-reactive antibodies. Science.

[bib45] Robbiani D.F., Bozzacco L., Keeffe J.R., Khouri R., Olsen P.C., Gazumyan A., Schaefer-Babajew D., Avila-Rios S., Nogueira L., Patel R. (2017). Recurrent potent human neutralizing antibodies to Zika virus in Brazil and Mexico. Cell.

[bib46] Robbiani D.F., Gaebler C., Muecksch F., Lorenzi J.C.C., Wang Z., Cho A., Agudelo M., Barnes C.O., Gazumyan A., Finkin S. (2020). Convergent antibody responses to SARS-CoV-2 in convalescent individuals. Nature.

[bib47] Saito T., Rehmsmeier M. (2015). The precision-recall plot is more informative than the ROC plot when evaluating binary classifiers on imbalanced datasets. PLoS One.

[bib48] Schatz D.G., Swanson P.C. (2011). V(D)J recombination: mechanisms of initiation. Annu. Rev. Genet..

[bib49] Scheid J.F., Barnes C.O., Eraslan B., Hudak A., Keeffe J.R., Cosimi L.A., Brown E.M., Muecksch F., Weisblum Y., Zhang S. (2021). B cell genomics behind cross-neutralization of SARS-CoV-2 variants and SARS-CoV. Cell.

[bib50] Schmitz A.J., Turner J.S., Liu Z., Zhou J.Q., Aziati I.D., Chen R.E., Joshi A., Bricker T.L., Darling T.L., Adelsberg D.C. (2021). A vaccine-induced public antibody protects against SARS-CoV-2 and emerging variants. Immunity.

[bib51] Setliff I., McDonnell W.J., Raju N., Bombardi R.G., Murji A.A., Scheepers C., Ziki R., Mynhardt C., Shepherd B.E., Mamchak A.A. (2018). Multi-donor longitudinal antibody repertoire sequencing reveals the existence of public antibody clonotypes in HIV-1 infection. Cell Host Microbe.

[bib52] Shang J., Wan Y., Luo C., Ye G., Geng Q., Auerbach A., Li F. (2020). Cell entry mechanisms of SARS-CoV-2. Proc. Natl. Acad. Sci. USA.

[bib53] Shiakolas A.R., Kramer K.J., Wrapp D., Richardson S.I., Schäfer A., Wall S., Wang N., Janowska K., Pilewski K.A., Venkat R. (2021). Cross-reactive coronavirus antibodies with diverse epitope specificities and Fc effector functions. Cell Rep. Med..

[bib54] Soto C., Bombardi R.G., Branchizio A., Kose N., Matta P., Sevy A.M., Sinkovits R.S., Gilchuk P., Finn J.A., Crowe J.E. (2019). High frequency of shared clonotypes in human B cell receptor repertoires. Nature.

[bib55] Soto C., Finn J.A., Willis J.R., Day S.B., Sinkovits R.S., Jones T., Schmitz S., Meiler J., Branchizio A., Crowe J.E. (2020). PyIR: a scalable wrapper for processing billions of immunoglobulin and T cell receptor sequences using IgBLAST. BMC Bioinformatics.

[bib56] Srivastava N., Hinton G., Krizhevsky A., Sutskever I., Salakhutdinov R. (2014). Dropout: a simple way to prevent neural networks from overfitting. J. Mach. Learn. Res..

[bib57] Starr T.N., Czudnochowski N., Liu Z., Zatta F., Park Y.J., Addetia A., Pinto D., Beltramello M., Hernandez P., Greaney A.J. (2021). SARS-CoV-2 RBD antibodies that maximize breadth and resistance to escape. Nature.

[bib58] Sui J., Hwang W.C., Perez S., Wei G., Aird D., Chen L.M., Santelli E., Stec B., Cadwell G., Ali M. (2009). Structural and functional bases for broad-spectrum neutralization of avian and human influenza A viruses. Nat. Struct. Mol. Biol..

[bib59] Tan T.J.C., Yuan M., Kuzelka K., Padron G.C., Beal J.R., Chen X., Wang Y., Rivera-Cardona J., Zhu X., Stadtmueller B.M. (2021). Sequence signatures of two public antibody clonotypes that bind SARS-CoV-2 receptor binding domain. Nat. Commun..

[bib60] Tareen A., Kinney J.B. (2020). Logomaker: beautiful sequence logos in Python. Bioinformatics.

[bib61] Thomson E.C., Rosen L.E., Shepherd J.G., Spreafico R., da Silva Filipe A., Wojcechowskyj J.A., Davis C., Piccoli L., Pascall D.J., Dillen J. (2021). Circulating SARS-CoV-2 spike N439K variants maintain fitness while evading antibody-mediated immunity. Cell.

[bib62] Tong P., Gautam A., Windsor I.W., Travers M., Chen Y., Garcia N., Whiteman N.B., McKay L.G.A., Storm N., Malsick L.E. (2021). Memory B cell repertoire for recognition of evolving SARS-CoV-2 spike. Cell.

[bib63] Tortorici M.A., Beltramello M., Lempp F.A., Pinto D., Dang H.V., Rosen L.E., McCallum M., Bowen J., Minola A., Jaconi S. (2020). Ultrapotent human antibodies protect against SARS-CoV-2 challenge via multiple mechanisms. Science.

[bib64] Trück J., Ramasamy M.N., Galson J.D., Rance R., Parkhill J., Lunter G., Pollard A.J., Kelly D.F. (2015). Identification of antigen-specific B cell receptor sequences using public repertoire analysis. J. Immunol..

[bib65] Vaswani A., Shazeer N., Parmar N., Uszkoreit J., Jones L., Gomez A.N., Kaiser L., Polosukhin I. (2017). 31st Conference on Neural Information Processing Systems (NIPS 2017).

[bib66] Voss W.N., Hou Y.J., Johnson N.V., Delidakis G., Kim J.E., Javanmardi K., Horton A.P., Bartzoka F., Paresi C.J., Tanno Y. (2021). Prevalent, protective, and convergent IgG recognition of SARS-CoV-2 non-RBD spike epitopes. Science.

[bib67] Walls A.C., Park Y.J., Tortorici M.A., Wall A., McGuire A.T., Veesler D. (2020). Structure, function, and antigenicity of the SARS-CoV-2 spike glycoprotein. Cell.

[bib68] Wang L., Zhou T., Zhang Y., Yang E.S., Schramm C.A., Shi W., Pegu A., Oloniniyi O.K., Henry A.R., Darko S. (2021). Ultrapotent antibodies against diverse and highly transmissible SARS-CoV-2 variants. Science.

[bib69] Wec A.Z., Wrapp D., Herbert A.S., Maurer D.P., Haslwanter D., Sakharkar M., Jangra R.K., Dieterle M.E., Lilov A., Huang D. (2020). Broad neutralization of SARS-related viruses by human monoclonal antibodies. Science.

[bib70] Wheatley A.K., Pymm P., Esterbauer R., Dietrich M.H., Lee W.S., Drew D., Kelly H.G., Chan L.J., Mordant F.L., Black K.A. (2021). Landscape of human antibody recognition of the SARS-CoV-2 receptor binding domain. Cell Rep..

[bib71] Winters A., McFadden K., Bergen J., Landas J., Berry K.A., Gonzalez A., Salimi-Moosavi H., Murawsky C.M., Tagari P., King C.T. (2019). Rapid single B cell antibody discovery using nanopens and structured light. mAbs.

[bib72] Wrapp D., Wang N., Corbett K.S., Goldsmith J.A., Hsieh C.L., Abiona O., Graham B.S., McLellan J.S. (2020). Cryo-EM structure of the 2019-nCoV spike in the prefusion conformation. Science.

[bib73] Wu N.C., Andrews S.F., Raab J.E., O'Connell S., Schramm C.A., Ding X., Chambers M.J., Leung K., Wang L., Zhang Y. (2020). Convergent evolution in breadth of two VH6-1-encoded influenza antibody clonotypes from a single donor. Cell Host Microbe.

[bib74] Wu N.C., Yamayoshi S., Ito M., Uraki R., Kawaoka Y., Wilson I.A. (2018). Recurring and adaptable binding motifs in broadly neutralizing antibodies to influenza virus are encoded on the D3-9 segment of the Ig gene. Cell Host Microbe.

[bib75] Wu N.C., Yuan M., Bangaru S., Huang D., Zhu X., Lee C.D., Turner H.L., Peng L., Yang L., Burton D.R. (2020). A natural mutation between SARS-CoV-2 and SARS-CoV determines neutralization by a cross-reactive antibody. PLoS Pathog..

[bib76] Ye J., Ma N., Madden T.L., Ostell J.M. (2013). IgBLAST: an immunoglobulin variable domain sequence analysis tool. Nucleic Acids Res..

[bib77] Yeap L.S., Hwang J.K., Du Z., Meyers R.M., Meng F.L., Jakubauskaitė A., Liu M., Mani V., Neuberg D., Kepler T.B. (2015). Sequence-intrinsic mechanisms that target AID mutational outcomes on antibody genes. Cell.

[bib78] Yu G. (2020). Using ggtree to visualize data on tree-like structures. Curr. Protoc. Bioinformatics.

[bib79] Yuan M., Liu H., Wu N.C., Lee C.D., Zhu X., Zhao F., Huang D., Yu W., Hua Y., Tien H. (2020). Structural basis of a shared antibody response to SARS-CoV-2. Science.

[bib80] Yuan M., Liu H., Wu N.C., Wilson I.A. (2021). Recognition of the SARS-CoV-2 receptor binding domain by neutralizing antibodies. Biochem. Biophys. Res. Commun..

[bib81] Zhang Q., Ju B., Ge J., Chan J.F., Cheng L., Wang R., Huang W., Fang M., Chen P., Zhou B. (2021). Potent and protective IGHV3-53/3-66 public antibodies and their shared escape mutant on the spike of SARS-CoV-2. Nat. Commun..

[bib82] Zhou B., Zhou R., Chan J.F.-W., Luo M., Peng Q., Yuan S., Mok B.W.-Y., Chen B., Wang P., Poon V.K.-M. (2022). An elite broadly neutralizing antibody protects SARS-CoV-2 Omicron variant challenge. Preprint at bioRxiv.

[bib83] Zhou P., Yang X.L., Wang X.G., Hu B., Zhang L., Zhang W., Si H.R., Zhu Y., Li B., Huang C.L. (2020). A pneumonia outbreak associated with a new coronavirus of probable bat origin. Nature.

[bib84] Zhou P., Yuan M., Song G., Beutler N., Shaabani N., Huang D., He W.T., Zhu X., Callaghan S., Yong P. (2022). A human antibody reveals a conserved site on beta-coronavirus spike proteins and confers protection against SARS-CoV-2 infection. Sci. Transl. Med..

